# Conservatism in Abdominal and Pelvic Surgery

**Published:** 1894-09

**Authors:** Floyd W. McRae

**Affiliations:** Atlanta, Ga.


### CONSERVATISM IN ABDOMINAL AND PELVIC SURGERY.*

By FLOYD W. McRAE, M.D.,

ATLANTA, GA.

In this discussion I have determined to confine my remarks to the subject of conservative surgery of the appendix. I do this because I have recently had some very interesting experiences with this most treacherous disease.

I consider that conservatism in medicine or surgery is pursuing the course that best conserves the life and health of the individual. I do not think it is conservatism to allow a patient to die without an operation when only an operation could save his life. It seems to me, however, that we have not yet reached a point where we can lay down the hard and fast rule that every case of appendicitis is an operative case. When men like Treves and other surgeons of equal eminence, claim that from eighty per cent. to ninety per cent. of all cases of appendicitis get well without operation, while autopsies show a pathological condition of the appendix in from sixteen per cent. to thirty per cent. without histories of attacks of appendicitis or evidence of recent trouble with the organ, it seems to me the time has not come when we may dogmatize.

*Read before Atlanta Society of Medicine, June 19, 1894.

Each case of appendicitis, however, must be considered from an operative standpoint, though many will not require surgical interference. I confess my inability to determine what to do in many instances. Surgeons of large experience make the same confession. I know of no better way to bring it home to the surgeon than after considering each case from every standpoint to then decide what course he would pursue if the patient were a member of his own family, or what he would want done if he were in a like condition. No conscientious surgeon could do more than that; humanity demands no less! It has seemed to me proper when the case is seen early to purge the bowels freely with salines, then, if there is no positive evidence of improvement in twenty-four hours, to advise immediate operation. Where the case is seen late, or the condition is critical, such a delay would prove fatal in many instances. I am speaking of acute appendicitis and not of "frequently recurring," or "chronic relapsing appendicitis."

By way of illustration I wish to present for your consideration brief histories of three very striking cases upon which I have operated in the last few weeks:

CASE I.—Julia H., aged twenty-two, single. No history of previous attacks. First called to see her Sunday, February 25. Found her complaining of pain and tenderness in right iliac region, temperature 101°, pulse 80. Prescribed salts in teaspoonful doses until bowels were freely purged. On Tuesday she came to my office and was feeling much better. I did not see her again until following Sunday, when I found her suffering very much as she was at my first visit one week before, and prescribed the same treatment. Monday she was no better, Tuesday she was much worse, and Wednesday her condition was extremely critical. Temperature 102°, pulse 140 to 150, abdomen immensely distended. Operation March 7th, incision, drainage, recovery. (Previously reported in a paper read before the Medical Association of Georgia.)

CASE II.—Jacob B., aged about twenty-eight, lawyer. History of repeated attacks of colic, and one or more of inflammation of bowels. First saw him in consultation with attending physician May 10th or 11th. Found him suffering with pain and tenderness in right iliac region, irregular temperature, ranging from 99° to

103°. His physician had diagnosed the case typhoid fever, and I was called to help determine whether he could go to his home fourteen miles in the country or not. I was not sure what his trouble was, but rather inclined to the idea that it was appendicitis. He was carried to his home, and I heard no more from him until the night of May 15th, when I was called to operate on him for rupture of the appendix and general peritonitis.

Operation, May 16th. Ether. Lateral incision; assisted by Drs. J. L. and A. A. Barge. Found appendix completely bound down by old and recent adhesions, slightly distended with very foul pus, beginning general peritonitis. Removed appendix, washed out abdominal cavity with hot water, inserted rubber and gauze drains, closed wound with silkworm gut sutures, and dressed in usual way. Strychnia and hot bottles were used freely to bring about reaction. Bowels were kept open with salts. Case progressed very favorbly until May 26th, when he was seized with violent cramps in bowels, requiring the hypodermatic injection of morphine to relieve it. From an oversight the bowels had not been moved for four or five days. Vomiting set in, and was so violent that everything taken into the stomach was rejected almost immediately. Impossible to move bowels.

Second operation May 28th. Assisted by Drs. J. L. and A. A. Barge, Kennedy and Hubbard. Chloroform. Patient *in extremis*. Reopened former incision with finger. Found complete paresis of bowels, adhesion of omentum to cecum and of several coils of small intestines to cecum and to each other. Broke up adhesions, packed wound with gauze and dressed in usual way. Vomiting continued. Gave strychnia, salts and elaterin hypodermatically, and succeeded in getting slight movement of bowels on May 31st, followed by free evacuations. His improvement had been steady, and he was convalescent on June 13th, when I last heard from him. (Recovery complete and uninterrupted.)

CASE III.—Walter W., aged sixteen, scrofulous diathesis. History of repeated attacks of stomach-ache, sometimes accompanied with fever. First called Sunday, June 17th. Found him with temperature of 101°, pulse 84, nauseated and vomiting occasionally, pain in epigastrium.

Prescribed saccharated calomel to be followed by saline. Called again next morning, found temperature 103°, pulse 96, pain and tenderness in the region of appendix very marked, muscles very tense, slight induration and dullness. Diagnosed appendicitis. Told family I thought an operation would be essential if he was not improved by next morning. Called Dr. Nicolson in consultation, and he agreed with diagnosis aud we put patient on teaspoonful doses of salts in hot water, to be repeated hourly until bowels were freely purged. Next morning, Tuesday, June 19th, temperature 103°, pulse 108. Operated at noon, assisted by Drs. Nicolson, Kennedy and Hubbard. Lateral incision. Found appendix very much enlarged, gangrenous and contained in an exceedingly thin-walled abscess, holding about four ounces of very dark, thin, fetid pus, which must have ruptured into general peritoneal cavity in a few hours. Removed appendix, inserted gauze and rubber drains, closed and dressed in usual way. Patient is apparently doing well, but as operation was only done to-day, cannot tell what the result will be. I have the appendix, which I pass around for your inspection. You see it is gangrenous at the tip and contains a concretion about the size of a plum seed.

[Case III. has had a very tedious recovery. He developed a fecal fistula on fourth day which discharged freely for about three weeks but closed spontaneously. Developed an abscess low down in pelvis fourth week, which was evacuated through original incision by tearing up adhesions with the finger. Ligature with which the appendix was tied off came away on thirty-sixth day. Patient now completely convalescent. Wound healed completely except small granulating spot where drainage was kept up for several weeks.]

For discussion of Dr. Olmsted's and Dr. McRae's papers see *Society Reports*.

